# Downstream Toll-like receptor signaling mediates adaptor-specific cytokine expression following focal cerebral ischemia

**DOI:** 10.1186/1742-2094-9-174

**Published:** 2012-07-16

**Authors:** Bolanle Famakin, Yongshan Mou, Maria Spatz, Modinat Lawal, John Hallenbeck

**Affiliations:** 1 Stroke Branch, National Institute of Neurological Disorders and Stroke, National Institutes of Health, 10 Center Drive, Building 10, Room 5B06, MSC 1401, Bethesda, MD 20892-1401, USA

**Keywords:** MyD88, TRIF, Focal ischemia, MCAO, Cytokines, TLR signaling, Toll-like, Receptor, Neutrophils, Leukocytes

## Abstract

**Background:**

Deletion of some Toll-like receptors (TLRs) affords protection against cerebral ischemia, but disruption of their known major downstream adaptors does not. To determine whether compensation in the production of downstream effectors by one pathway when the other is disrupted can explain these findings, we examined cytokine/chemokine expression and inflammatory infiltrates in wild-type (WT), MyD88^−/−^ and TRIF-mutant mice following permanent middle cerebral artery occlusion (pMCAO).

**Methods:**

Cytokine/chemokine expression was measured with a 25-plex bead array in the serum and brains of all three groups of mice at baseline (no surgery/naïve) and at 3 hours and 24 hours following pMCAO. Brain inflammatory and neutrophil infiltrates were examined 24 hours following pMCAO.

**Results:**

IL-6, keratinocyte chemoattractant (KC), granulocyte colony-stimulating factor (G-CSF) and IL-10 were significantly decreased in MyD88^−/−^ mice compared to WT mice following pMCAO. Significantly, decreased levels of the neutrophil chemoattractants KC and G-CSF corresponded with a trend toward fewer neutrophils in the brains of MyD88^−/−^ mice. IP-10 was significantly decreased when either pathway was disrupted. MIP-1α was significantly decreased in TRIF-mutant mice, consistent with TRIF-dependent production. Finally, MyD88^−/−^ mice showed elevations of a number of Th2 cytokines, such as IL-13, at baseline, which became significantly decreased following pMCAO.

**Conclusions:**

Both MyD88 and TRIF mediate pathway-specific cytokine production following focal cerebral ischemia. Our results also suggest a compensatory Th2-type skew at baseline in MyD88^−/−^ mice and a paradoxical switch to a Th1 phenotype following focal cerebral ischemia. Finally, the MyD88 pathway directs the expression of neutrophil chemoattractants following cerebral ischemia.

## Background

Stroke remains a leading cause of death and disability in many parts of the world, including the United States, and approved therapies are currently limited. At the cellular level, a number of mechanisms, including inflammation, play a key role in the pathogenesis of a host of neurological diseases and acute cerebral ischemia. Therefore, a potential approach to therapy includes attenuating the inflammatory damage that results from cerebral ischemia. In this regard, it is important to study and understand key signal transduction elements in pathways such as the Toll-like receptor (TLR) signaling pathway that lead to activation of major transcriptional mediators of inflammation (,i.e. NF-κB and activator protein 1) [1].

The TLR signaling pathway is evolutionarily conserved across several species and was initially identified as important in the immune response to infection. Subsequently, the TLR signaling pathway has been shown to play a pivotal role in the inflammatory response following ischemia-reperfusion in several organ systems [2,3].

TLRs recognize both exogenous pathogen-associated molecular patterns (PAMPs) on infectious organisms and endogenous danger-associated molecular patterns (DAMPs) associated with tissue stress or injury [4,5]. The role of different TLRs in cerebral ischemia has been studied extensively [6-8], but the accompanying downstream TLR signaling events remain poorly understood. Recently, we found no significant differences in infarct size or neuron survival in models of focal, global or *in vitro* ischemia in mice with deletions or mutations of the downstream TLR signaling adaptor molecules Myeloid-dependent protein-88 (MyD88) and Toll/interleukin 1 receptor domain-containing adaptor-inducing interferon β (TRIF). In contrast, interestingly, assessment of infarct sizes showed trends toward being larger in MyD88^−/−^ and TRIF-mutant mice (hereafter referred to as TRIF) compared to wild type (WT) following focal ischemia [9].

Furthermore, our previous results, as well as those of others, suggest that downstream TLR signaling may be organ-specific: different in the brain compared to the periphery [9-12]. As a result, these studies were further extended to characterize the cytokine production profile in response to downstream TLR signaling following cerebral ischemia. We studied the cytokine/chemokine profile via the MyD88-independent (TRIF) pathway and the MyD88-dependent pathway, which mediates downstream TLR signaling via all TLRs except TLR3. This was evaluated by simultaneous determination of the levels of 25 cytokines and chemokines in the brains and sera of MyD88^−/−^ and TRIF-mutant compared to WT mice. Cytokine and chemokine analysis was performed at baseline and at different time points following focal cerebral ischemia. To complement our current studies, we also determined, in parallel, the inflammatory infiltrate in the brains of mice subjected to permanent middle cerebral artery occlusion (pMCAO). The evaluation of inflammatory infiltrate was performed at 24 hours, since neutrophils have been shown to be abundant at this time point in models of murine pMCAO as used in our study [13,14].

In the present article, we report that cerebral ischemia leads to specific downstream adaptor-dependent production of cytokines and chemokines. In addition, importantly, our present studies show that specific downstream TLR adaptors control the secretion of neutrophil chemoattractant chemokines that direct the migration of neutrophils to the site of ischemia. Interestingly, we also demonstrate that mice with disruption of one of the major downstream signaling TLR pathways have an unexpected shift in network dynamics those results in a specific baseline cytokine profile that may be maladaptive toward the innate immune response to cerebral ischemia. Overall, these studies provide in-depth and novel insights into the complex downstream TLR signaling events following focal cerebral ischemia.

## Materials and methods

### Animals

The inclusion criteria for the mice in our MCAO studies were (1) male, (2) ages 12 to 16 weeks old and (3) no obvious signs of infection. Female mice, mice younger than 12 weeks or older than 16 weeks of age, and mice with signs of bite wounds or other signs of infection were excluded from the study. Mice were selected at random from cages for inclusion in the study. Breeder pairs for MyD88^−/−^ mice were obtained from the Institute for Systems Biology, Seattle, WA, USA. MyD88^−/−^ mice (hereafter referred to as MyD), have deletions in exons IV and V encoding the carboxyl terminal portion of the *MyD88* gene, which contains the Toll/interleukin 1 receptor (TIR) domain required for downstream signaling [15]. WT C57BL/6J and TRIF-mutant mice were obtained from The Jackson Laboratory (Bar Harbor, ME, USA). TRIF-mutant mice have a single base pair deletion predicted to remove 24 amino acids from the carboxyl terminus of the protein [16]. Average weights for animals were as follows: WT, 28.7 ± 2.5 g; MyD, 29.5 ± 2.4 g; and TRIF, 27.1 ± 1.6 g. All mice were housed and bred at the National Institute of Neurological Disorders and Stroke (NINDS) animal care facility under 12-hour light and dark cycles. Animals had free access to food and water both pre- and post surgery. All animal procedures were approved by the NINDS Animal Care and Use Committee.

### Focal ischemia

pMCAO was carried out as previously described [9]. Briefly, anesthesia was induced and maintained with isoflurane and the distal left MCAO was cauterized by means of an electrocoagulator (ICC 200; ERBE Elektromedizin, Tübingen, Germany). Rectal temperature was maintained at 37°C in mice from all three groups during surgery by using a heating pad. No mice died during or after MCAO surgery. The surgeon who performed the MCAO surgery (YM) was blinded to the genotype of the animal. Mouse tails were color-coded for identification (by BF) during pMCAO surgery. One MyD88^−/−^ mouse was excluded from cytokine analysis because of hemorrhage during MCAO surgery. The decision to exclude this animal from analysis was made by the surgeon, who was unaware of the group to which the animal belonged. Euthanasia was carried out with lethal doses of isoflurane and nitrous oxide followed by bilateral pneumothorax.

### Experimental groups

#### Cytokine/chemokine analysis

Twenty-seven mice were used for brain and serum cytokine/chemokine analysis, comprising nine per group for WT, TRIF and MyD88^−/−^. Each group of nine mice consisted of three mice each without surgery (baseline) three mice at 3 hours and three mice at 24 hours following pMCAO.

### Immunohistochemistry

Immunohistochemical studies were performed 24 hours following pMCAO. There were a total of nine mice: three per group for WT, TRIF-mutant and MyD88^−/−^ mice.

### Sample preparation for cytokine/chemokine analysis

#### Serum

Mice were euthanized at the above-mentioned time points. Whole blood was collected via cardiac puncture, transferred into serum gel microtubes (SARSTEDT AG & Co, Nümbrecht, Germany) and allowed to coagulate at room temperature for at least 20 minutes before centrifugation at approximately 6,000 *g* for 10 minutes. The separated serum was stored at −80°C until further analysis.

### Brain homogenate

At the time points indicated above, the same mice from which serum had been collected were euthanized, and the subcortical structures were removed from ischemic and non-ischemic cortices and stored at −80°C until analysis. Subsequently, brain tissues were homogenized in ice-cold 1× phosphate-buffered saline (PBS) (pH 7.4), containing 1× protease inhibitor cocktail (Sigma-Aldrich, St Louis, MO, USA) and 0.2% Triton X-100 (Sigma-Aldrich), for 30 seconds, using an IKA T 10 basic homogenizer (IKA Works, Wilmington, NC, USA). The resulting homogenates were centrifuged at 15,000 *g* for 30 minutes, at 4°C, and supernatants were stored at −80°C until cytokine analysis.

### Cytokine determination by 25-plex magnetic bead panel

Serum and extracts from homogenized brains were analyzed simultaneously for levels of 25 mouse cytokines and chemokines using the Milliplex MAP 25-plex premixed mouse cytokine/chemokine magnetic bead panel (Millipore), according to manufacturer’s instructions. The 25 cytokines and chemokines analyzed included G-CSF, granulocyte macrophage colony-stimulating factor, IFN-γ, IL-1α, IL-1β, IL-2, IL-4, IL-5, IL-6, IL-7, IL-9, IL-10, IL-12 (p40), IL-12 (p70), IL-13, IL-15, IL-17, inducible protein 10 (IP-10), KC, monocyte chemoattractant protein 1 (MCP-1), macrophage inflammatory protein 1α (MIP-1α), MIP-1β, MIP-2, RANTES (regulated upon activation, normal T-cell expressed, and secreted) and TNF-α.Briefly, 25 μl of premixed magnetic beads conjugated to antibodies for all 25 analytes were added to each well of a 96-well, flat-bottomed plate containing 25 μl of brain or serum samples and diluted 1:2 in assay buffer. Plates were protected from light and incubated on an orbital shaker for 2 hours at room temperature. Incubation was followed by a wash step (three times with 200 μl of wash buffer) on a magnetic plate washer. Detection antibodies (25 μl) were added to each well, and plates were reincubated with agitation for 1 hour at room temperature. Streptavidin-phycoerythrin (25 μl) was added to each well, and plates were reincubated with agitation for another 30 minutes, followed by a final wash step. Magnetic beads were resuspended in sheath fluid and assayed on a Luminex 100 xMAP technology machine (Luminex, Austin, TX, USA) using Bioplex Manager 6.0 software. Standard curves were generated for each cytokine/chemokine using standards included in each kit. The median fluorescence intensity for each analyte was calculated using a four- or five-point logistic parameter curve. Chemokine/cytokine levels below the level of detection of the assay were calculated using a default value of 0 pg/ml for that particular analyte.

### Immunohistochemistry

Brains were removed from a separate group of mice for immunohistochemical studies 24 hours after pMCAO, quickly frozen on dry ice and stored at −80°C until further analysis. Brains sections (16 μm) were fixed with 2% buffered formaldehyde (Mallinckrodt Chemical Co, St Louis, MO, USA) in PBS, at 4°C, for 8 minutes. Endogenous peroxidase was quenched using hydrogen peroxide (0.3%) in methanol for 20 minutes. Bovine serum albumin (Sigma-Aldrich) and 10% normal serum from the originating species of the secondary antibody were used for blocking. Subsequently, blocked sections were incubated with primary antibody overnight for 18 to 24 hours at 4°C and with secondary antibody at room temperature for up to 1.5 hours. Immunoreactivity with primary antibody was visualized with avidin-biotin staining (ABC kit; Vector Laboratories, Burlingame, CA, USA) and diaminobenzene (DAB) staining (Vector Laboratories). Primary antibodies used were anti-Ly6B.2 (cat. no. MCA771GA, 2 μg/ml; AbD Serotecand anti-CD45 (cat. no. AF114, 2 μg/ml; R&D Systems, Minneapolis, MN, USA) for neutrophils and leukocytes, respectively. Secondary antibodies included biotin-conjugated donkey anti-rat (cat. no. 712-066-153) and donkey anti-goat (cat. no. 705-066-147) at 1:3,000 concentrations (both from Jackson Immunoresearch Laboratories, West Grove, PA, USA) for anti-Ly6B.2 and anti-CD45, respectively.

Nonspecific immunoreactivity with primary antibodies was determined by the lack of DAB staining in negative controls incubated with normal immunoglobulin G derived from the species of the primary antibody. Specific antibody immunoreactivity was determined by the presence of DAB staining in stained sections incubated with primary antibody. Primary antibody immunoreactivity in sections from WT mice were compared to those from MyD88^−/−^ and TRIF-mutant mice brain sections. The number of neutrophils or leukocytes per high-powered field (10×) were counted by two independent investigators (BF and ML), and the averages of these counts were used for data analysis.

### Statistical analysis

Three mice per time point and nine animals per group were used in MCAO studies to obtain statistically analyzable data on variability with regard to cytokine production following pMCAO among the different groups of mice. Comparison of means among experimental groups was done using the nonparametric Kruskal-Wallis method (StatView version 5.0 software; SAS Institute, Cary, NC, USA). Nonparametric Mann–Whitney *U* statistics was used for comparison of means between two groups. Differences among groups were expressed as means ± SD and considered significant if *p* < 0.05.

## Results

### MyD88-dependent cytokines/chemokines in brain and serum following pMCAO

**Figure 1:**
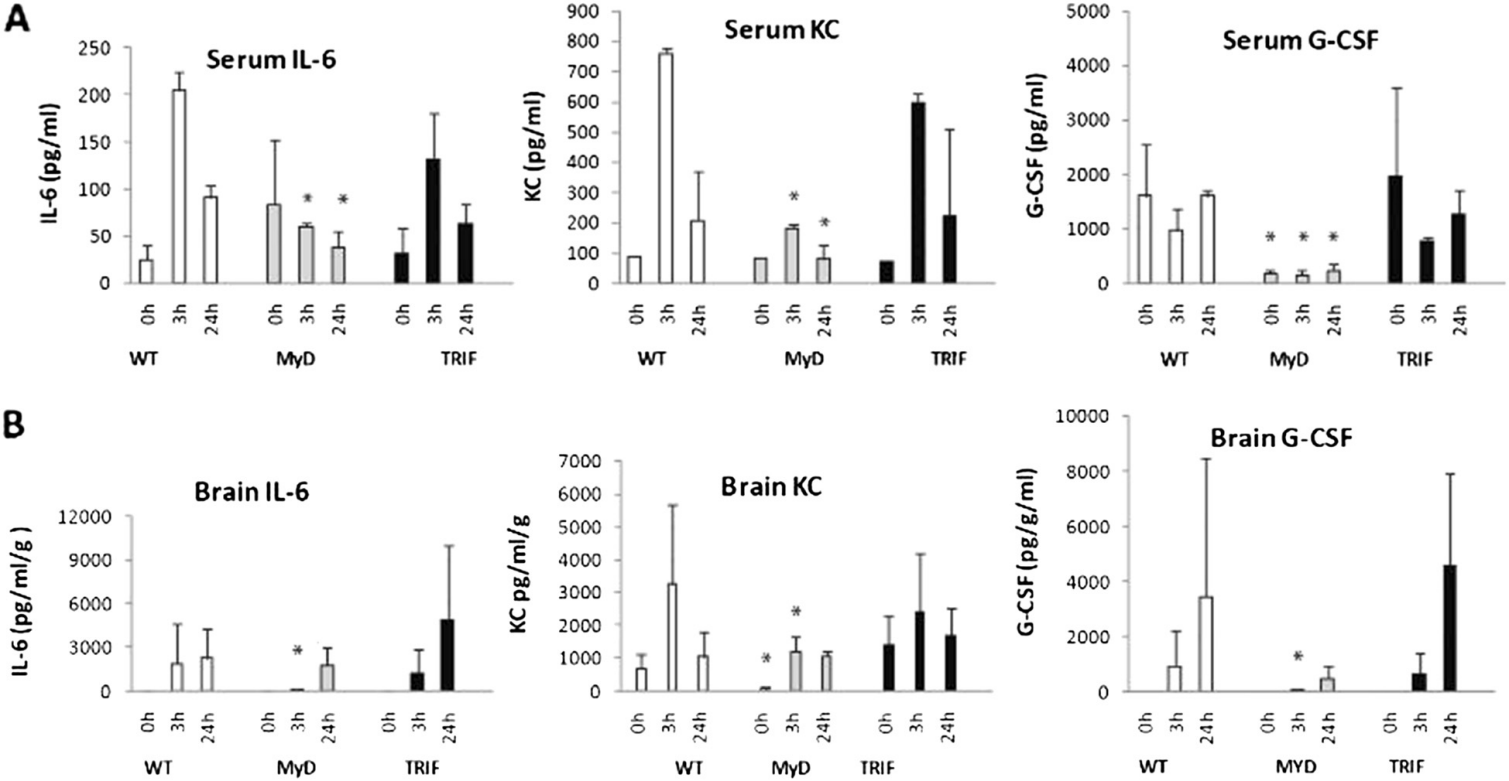
**MyD88-dependent cytokines following permanent middle cerebral artery occlusion.** Significantly decreased levels of IL-6 and neutrophil chemoattractants keratinocyte chemoattractant and granulocyte colony-stimulating factor in MyD88^−/−^ mice compared to wild type (WT) at the indicated time points in serum **(A)** and brain **(B)**. ^*^p < 0.05, MyD88^−/−^ compared to WT. *N* = 9 per group and *n* = 3 per time point. 0 hours = baseline.

IL-6, KC and G-CSF were all produced in a MyD88-dependent manner in the serum, as illustrated by significantly decreased levels of these cytokines/chemokines in MyD88^−/−^ mice compared to WT mice following pMCAO (Table 1 and Figure 1A). The same pattern was seen in the brain, where the levels of these same cytokines and chemokines were significantly attenuated in MyD88^−/−^ mice compared to WT mice following focal cerebral ischemia (Table 1 and Figure 1B). Interestingly, however, the significant decreases in the levels of these MyD88^−/−^-dependent cytokines/chemokines occurred in spite of abnormal elevations, at baseline, of cytokines such as IL-6 in the serum of MyD88^−/−^ mice, regardless of whether the cytokine/chemokine in question increased or decreased with ischemia (Figure 1A). Levels of chemokines such as KC were comparable at baseline in all three groups of mice, but significantly decreased in MyD88^−/−^ mice, compared to WT mice following pMCAO, indicative of MyD88-dependent production of this chemokine (Figure 1A). In addition, of interest, some chemokines, such as G-CSF, were significantly decreased in serum at baseline in MyD88^−/−^ mice and also significantly decreased compared to WT with ischemia (Figure 1A). Furthermore, of note, serum G-CSF levels decreased in the serum following pMCAO at the same time point at which they were increasing in the brain following focal ischemia (Figures 1A and 1B).

**Table 1:** MyD88-dependent cytokines/chemokines following permanent middle cerebral artery occlusion

**Location**		**WT**		**MyD88**^**−/−**^		**TRIF**		** *p* -value**
Serum (pg/ml)								
IL-6	Baseline	24.7 ± 15.2		83.3 ± 69.5		30.9 ± 28.7		n.s.^†^
	3 hours	205.2 ± 19.8	(↑8.3)	59.9 ± 3.9	(↓1.4)	130.9 ± 50.7	(↑4.2)	0.027,^*^ <0.05^†^
	24 hours	91.5 ± 12.6		37.3 ± 18.7		62.9. ± 21.0		<0.05^†^
KC	Baseline	88.5 ± 19.4		81.1 ± 12.3		70.3 ± 29.9		n.s.^†^
	3 hours	758.8 ± 164.4	(↑8.6)	182.5 ± 47.9	(↑2.3)	595.1 ± 291.3	(↑8.5)	<0.05^†^
	24 hours	208.1 ± 52.6		81.3 ± 21.1		221.3 ± 103.3		<0.05^†^
G-CSF	Baseline	1,613.8 ± 939.8		185.7 ± 54.6		1,965.3 ± 1,626.3		<0.05^†^
	3 hours	980.3 ± 424.0	(↓1.6)	154.3 ± 88.6	(↓1.2)	787.4 ± 61.5	(↓2.5)	<0.05^†^
	24 hours	1,566.7 ± 91.0		207.8 ± 133.8		1,282.7 ± 444.9		<0.05^†^
Brain (pg/g/ml)								
IL-6	Baseline	ND		ND		ND		
	3 hours	1,912.3 ± 2,665.9		182.3 ± 54.4		1,269.8 ± 1,643.7		<0.05^†^
	24 hours	2,293.7 ± 1,986.5		1,811.9 ± 1,224.0		4,858.6 ± 5201.7		n.s.
KC	Baseline	692.9 ± 461.5		108.2 ± 15.7		1,400.3 ± 879.4		0.030,^*^ 0.034^†^
	3 hours	3,271.3 ± 2,446.9	(↑4.7)	1,230.1 ± 426.6	(↑11.4)	2,405.8 ± 1,823.6	(↑1.7)	<0.05^†^
	24 hours	1,066.0 ± 760.4		1,080.3 ± 168.6		1,694.2 ± 836.7		n.s.
G-CSF	Baseline	16.7 ± 14.4		3.3 ± 3.8		14.2 ± 7.7		n.s.
	3 hours	962.5 ± 1,272.7	(↑57.6)	57.8 ± 26.3	(↑17.5)	652.5 ± 749.6	(↑45.9)	<0.05^†^
	24 hours	3,458.4 ± 5,056.6		505.3 ± 480.5		4,610.8 ± 3,333.3		n.s.
IL-10	Baseline	91.3 ± 85.7		195.2 ± 204.9		510.9 ± 447.3		n.s.
	3 hours	1,047 ± 621.76	(↑11.5)	87 ± 55.25	(↓2.2)	319.1 ± 415.77	(↓1.6)	<0.05^†^

*G-CSF* granulocyte colony-stimulating factor, *IL* interleukin, *KC* keratinocyte chemoattractant, *ND* not detectable, *TRIF* Toll/interleukin 1 receptor domain-containing adaptor-inducing interferon β, *WT* wild type.

^*^Significantly different between all three groups using Kruskal-Wallis test.

^†^Significantly different between WT and MyD88 using Mann–Whitney test.

Parentheses: Fold change compared to baseline for that particular group.

On the other hand, in TRIF-mutant mice, levels of IL-6, KC and G-CSF were comparable to the levels and expression patterns of these chemokines in WT mice in serum and brain following pMCAO. These findings are consistent with MyD88-dependent production of these chemokines following pMCAO (Table 1 and Figure 1).

Brain IL-10 levels were significantly decreased in MyD88^−/−^ mice compared to WT 3 hours following pMCAO, consistent with MyD88-dependent production of this cytokine (Table 1).

**Figure 2:**
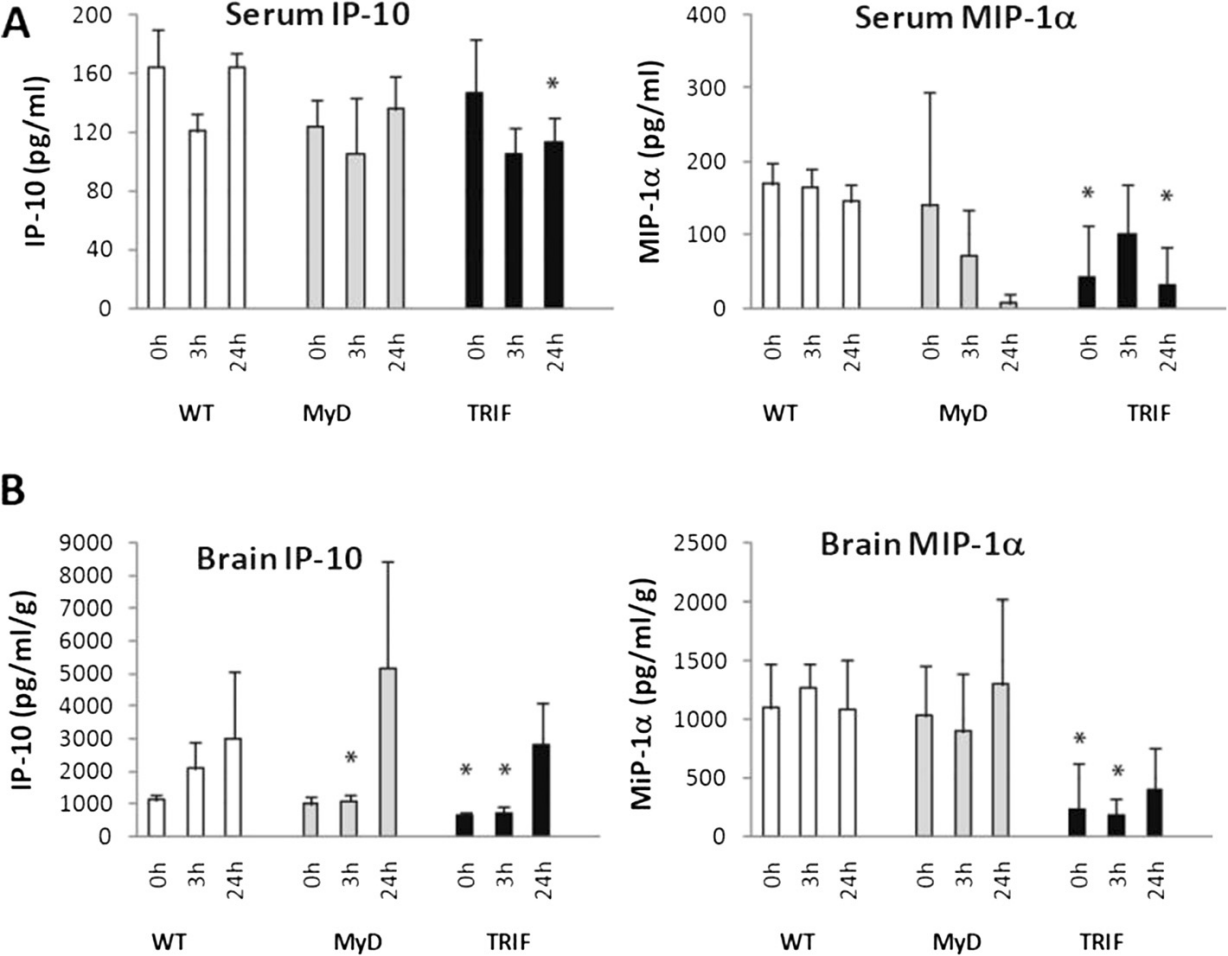
**Toll/interleukin 1 receptor domain-containing adaptor-inducing interferon β (TRIF)-dependent cytokines following permanent middle cerebral artery occlusion.** Significantly decreased levels of inducible protein 10 (IP-10) and macrophage inflammatory protein 1α (MIP-1α) in TRIF-mutant compared to wild type (WT) at the indicated time points in serum **(A)** and brain **(B)** following permanent middle cerebral artery occlusion (pMCAO). IP-10 levels are also significantly decreased in MyD88^−/−^ mice at 3 hours following permanent middle cerebral artery occlusion. ^*^p < 0.05, MyD88^−/−^ or TRIF compared to WT. *N* = 9 per group and *n* = 3 per time point. 0 hours = baseline.

Another chemokine that seemed to be dependent, in part, on the MyD88 pathway in the brain following pMCAO was IP-10. Levels of this chemokine were significantly decreased in the brains of MyD88^−/−^ mice at 3 hours following pMCAO (Figure 2). In the periphery, however, production of this chemokine was more dependent on the TRIF pathway following pMCAO (Table 2 and Figure 2A).

**Table 2:** TRIF-dependent cytokine/chemokines following permanent middle cerebral artery occlusion

**Location**		**WT**	**MyD88**^**−/−**^	**TRIF**	** *p* -value**
Serum (pg/ml)					
IP-10	Baseline	164.8 ± 25.1	124.1 ± 17.6	147.5 ± 35.9	n.s.
	3 hours	121.4 ± 11.4	105.9 ± 37.0	104.9 ± 18.9	n.s.
	24 hours	164.7 ± 9.1	135.8 ± 21.9	113.9 ± 16.7	<0.05^†^
MIP-1α	Baseline	169.3 ± 28.1	140.9 ± 153.9	41.7 ± 72.2	<0.05^†^
	3 hours	166.4 ± 23.4	71.5 ± 62.7	100.4 ± 68.3	n.s.
	24 hours	145.7 ± 22.6	6.7 ± 11.6	30.9 ± 53.5	<0.05^†^
Brain (pg/ml/g)					
IP-10	Baseline	1,095.9 ± 159.5	985.4 ± 207.7	619.2 ± 141.2	<0.05^†^
	3 hours	2,077.2 ± 803.6	1087.5 ± 190.4	710.5 ± 223.0	0.039,^*^ <0.05^†^
	24 hours	2,997.3 ± 2,074.2	5169.9 ± 3305	2,820 ± 1,273	n.s.
MIP-1α	Baseline	1,096.3 ± 372.6	1026.1 ± 434.6	228.2 ± 395.3	<0.05^†^
	3 hours	1,267.8 ± 210.9	891.5 ± 501.6	171.2 ± 148.7	<0.05^†^
	24 hours	1,088.2 ± 413.6	1293.9 ± 741.1	403.1 ± 355.7	n.s.

*IP-10* inducible protein 10, *MIP-1α* macrophage inflammatory protein 1α, *TRIF* Toll/interleukin 1 receptor domain-containing adaptor-inducing interferon β, *WT* wild type.

^*^Significantly different between all three groups using Kruskal-Wallis.

^†^Significantly different between WT and TRIF mutant using Mann–Whitney test.

Parentheses: fold change compared to baseline for that particular group.

### TRIF-dependent cytokines/chemokines in the brain and serum following pMCAO

IP-10 production in the brain was significantly decreased in mice with disruption of either TRIF or MyD88 at 3 hours following pMCAO. This suggests that both of these pathways contribute to the production of IP-10 following focal cerebral ischemia (Figure 2A and 2B). However, brain levels of IP-10 were significantly lower in TRIF-mutant mice at both baseline and 3 hours following pMCAO. In addition, serum IP-10 levels were significantly decreased in TRIF-mutant mice at 24 hours following focal ischemia. These combined findings indicate possible tissue specificity by these adaptors and a greater contribution from the TRIF pathway in IP-10 production (Figures 2A and 2B and Table 2). In contrast, MIP-1α was clearly produced in a predominantly TRIF-dependent manner following pMCAO. MIP-1α levels were significantly decreased at baseline in TRIF-mutant mice in both the brain and serum, and subsequently at specific time points, following focal cerebral ischemia. Specifically, MIP-1α levels were significantly decreased at 3 hours in the brain and at 24 hours in the serum compared to WT following pMCAO (Table 2 and Figures 2A and 2B).

### Th2-associated cytokines in MyD88^−/−^ mice compared to baseline following pMCAO

**Figure 3:**
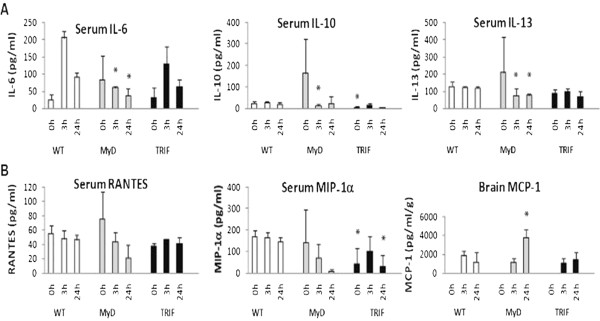
**Th2 cytokines at baseline. (A)** Abnormal elevations in levels of Th2 cytokines IL-6, IL-10 and IL-13 and **(B)** Th2-associated chemokines, RANTES (regulated upon activation, normal T-cell expressed, and secreted) and macrophage inflammatory protein 1α (MIP-1α), in MyD88^−/−^ mice at baseline and significant decreases in levels of these cytokines at the indicated time points following permanent middle cerebral artery occlusion (pMCAO) compared to wild type (WT). Interestingly, Th1-associated monocyte chemoattractant protein 1 significantly increased in the brain of MyD88^−/−^ mice 24 hours following pMCAO when levels have started to decrease towards baseline in WT mice. *p < 0.05; MyD88^−/−^ or TRIF-mutant compared to WT. *N* = 9 per group and *n* = 3 per time point. 0 hours = baseline.

Levels of Th2 cytokines such as IL-13 were significantly decreased in MyD88^−/−^ mice (*p* < 0.05) compared to WT at 3 and 24 hours following focal cerebral ischemia (Figure 3). This decrease occurred in the context of apparently elevated levels of these cytokines and their associated chemokines, such as RANTES and MIP-1α, at baseline in MyD88^−/−^ mice (Figures 3A and 3B). The expression of other Th2 cytokines, such as IL-6 and IL-10, as well as Th2-associated chemokines, RANTES and MIP-1α, followed the same pattern. The expression pattern of these Th2 cytokines in MyD88^−/−^ mice is consistent with a switch to a more Th1 type phenotype following focal cerebral ischemia. Also consistent with switching to a more Th1 phenotype was a significant increase in Th1 cytokines, such as MCP-1 (*p* < 0.05), in the brains of MyD88^−/−^ mice at 24 hours, when levels had started to decrease back to baseline in WT mice (Figure 3B).

### Inflammatory infiltrate in the brains of wild-type, MyD88^−/−^ and TRIF-mutant mice following pMCAO

#### Leukocytes

**Figure 4:**
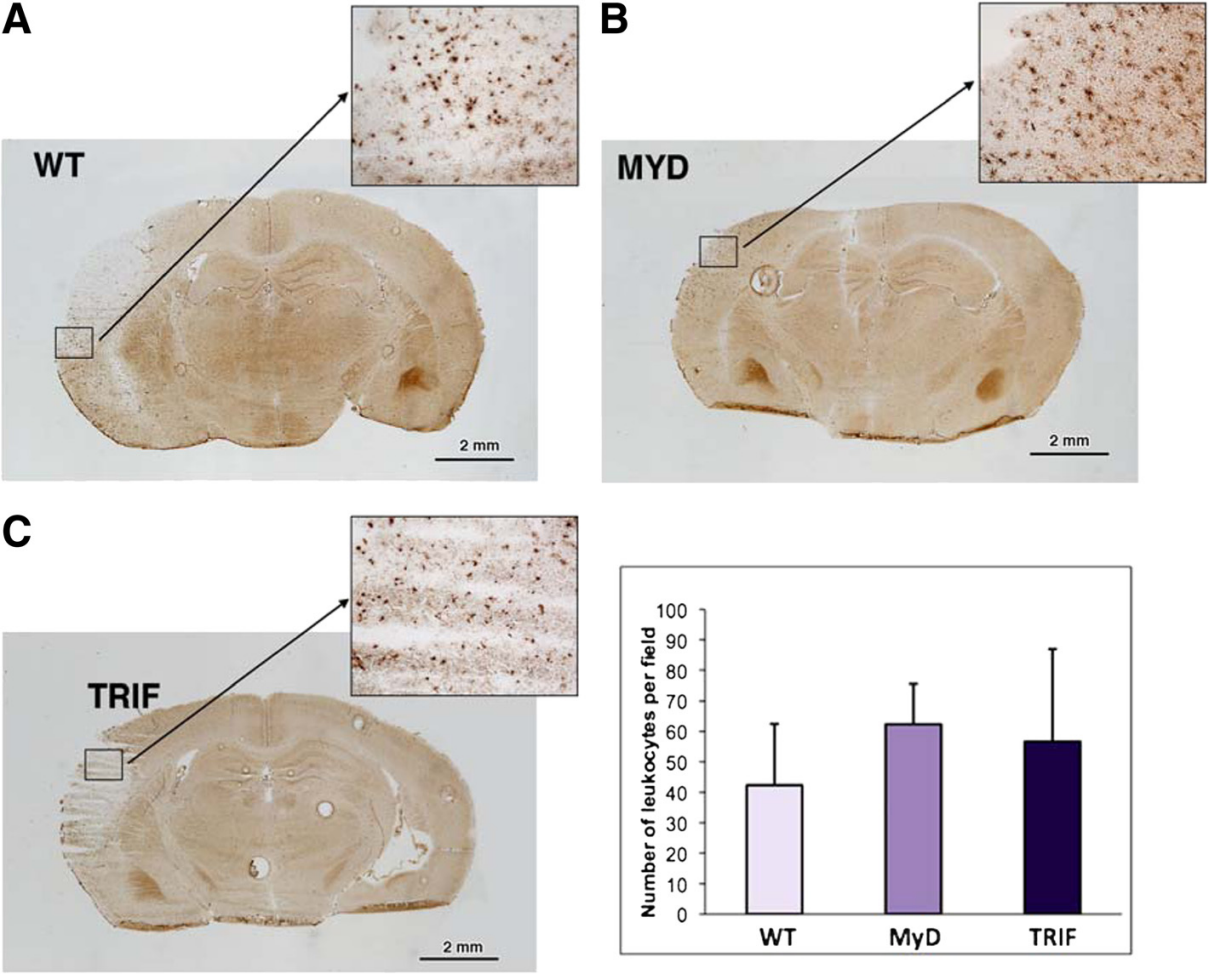
**Leukocyte infiltrate in the brains of (A) wild-type, (B) MyD88**^**−/−**^**and (C) TRIF-mutant mice 24 hours following permanent middle cerebral artery occlusion.** Inset: Graph shows the number of leukocytes per high-powered field (10×) in each group of mice. There were no differences in leukocyte infiltrate in the brains of all three groups of mice at 24 hours following focal ischemia (*p* = 0.3607). *N* = 9 mice, and *n* = 3 mice per wild type, MyD88^−/−^ and TRIF-mutant.

Leukocyte numbers per high-powered field (10×), were not significantly different among brains from all three groups of mice: WT, 42.3 ± 20.1; MyD, 62.3 ± 13.2; and TRIF-mutant, 56.7 ± 30.3 (*p* = 0.3607). At 24 hours following pMCAO, robust leukocyte infiltration was observed in brains from all three groups of mice, as shown in Figures 4A, 4B and 4C for WT, MyD and TRIF-mutant mice, respectively.

#### Neutrophils

**Figure 5:**
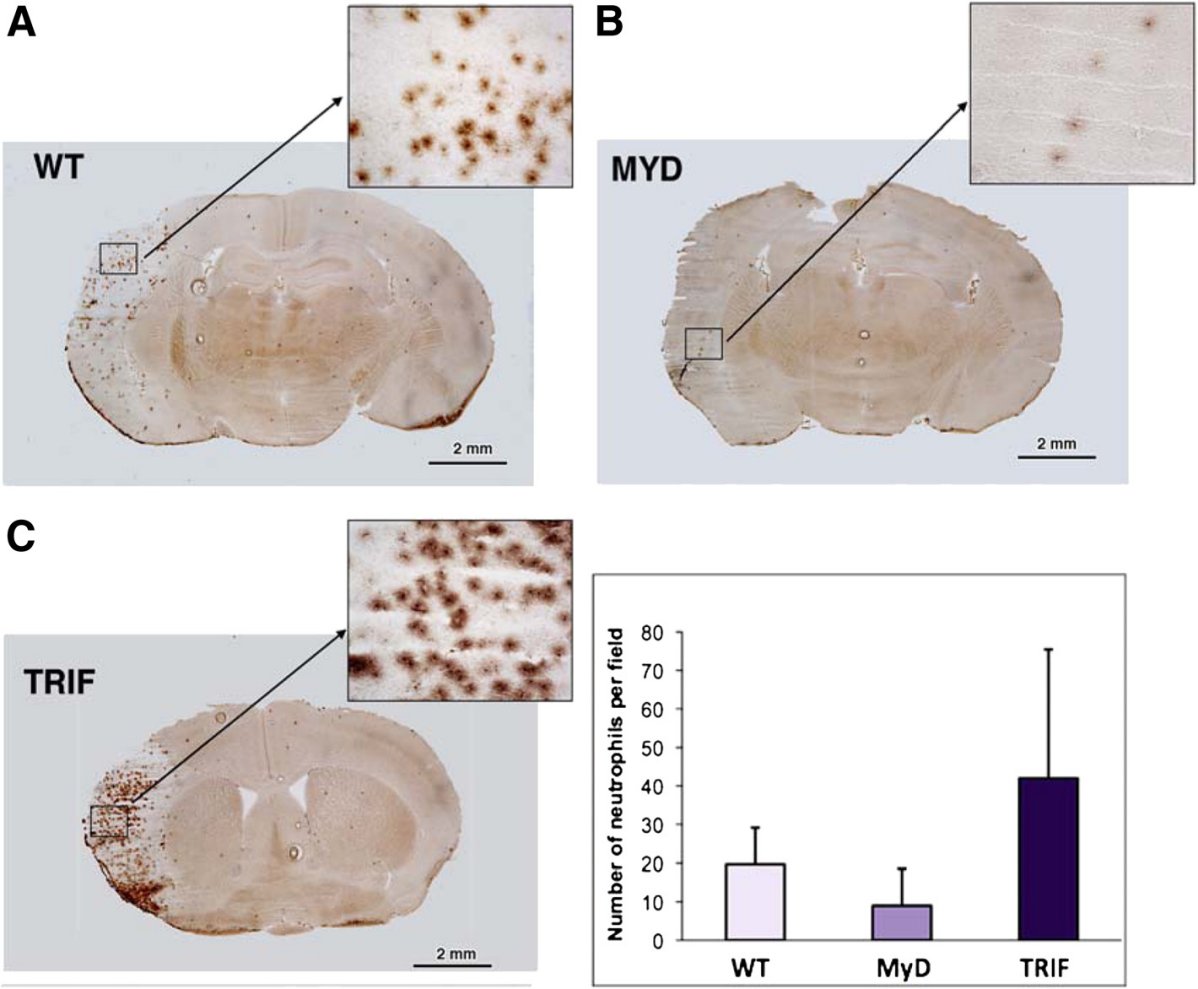
**Neutrophil infiltrate in the brains of (A) wild-type, (B) MyD88**^**−/−**^**and (C) TRIF-mutant mice at 24 h following permanent middle cerebral artery occlusion.** Inset: Graph shows the number of neutrophils per high-powered field (10×) in each group of mice. There was a trend toward fewer neutrophils in the brains of MyD88^−/−^ mice compared to wild-type (WT) and Toll/interleukin 1 receptor domain-containing adaptor-inducing interferon β (TRIF)-mutant mice at 24 hours following permanent middle cerebral artery occlusion (*p* = 0.189). *N* = 9 and *n* = 3 mice per WT, MyD88^−/−^ and TRIF-mutant.

The brains of MyD88^−/−^ mice showed fewer neutrophils per high-powered field (10×): WT, 17.3 ± 8.0; MyD88^−/−^, 8.3 ± 9.3; and TRIF-mutant, 35.2 ± 24.3 (*p* = 0.149). In contrast to the findings in MyD88^−/−^ mice, we observed the opposite pattern in the brains of TRIF-mutant mice, in which the predominant functioning pathway is the MyD88 pathway. Figures 5A, 5B and 5C show neutrophil infiltration in the brains of WT, MyD and TRIF-mutant mice, respectively.

## Discussion

This study demonstrates a distinct pattern of cytokine/chemokine production following focal ischemia in mice with disruptions of MyD88 and TRIF. It also shows that the MyD88 pathway plays a major role in the production of neutrophil chemoattractants following focal ischemia. Patterns of cytokine production in response to ischemia are altered in mice with disruptions of these downstream adaptors. These findings indicate that cytokine/chemokine production following focal cerebral ischemia is mediated by these downstream TLR signaling pathways. Our results also suggest a possible compensatory skew toward the Th2 adaptive immune response at baseline in mice with disruptions of MyD88 and a subsequent paradoxical Th1 switch following focal cerebral ischemia. These findings support our previous supposition that the lack of protection against cerebral ischemia noted in MyD88^−/−^ and TRIF-mutant mice was not due to compensation in the production of downstream effectors by either pathway when the other was disrupted [9].

Serum and brain IL-6 production during focal cerebral ischemia followed previously reported kinetics [17,18]. There was no apparent compensation in IL-6 levels by the TRIF pathway. Overall, our current results demonstrate that IL-6 production is MyD88-dependent following focal ischemia. IL-6 levels were similar in mice with disruptions of the TRIF pathway and WT mice. These findings explain, in part, our earlier results which showed that MyD88^−/−^ mice were not protected against cerebral ischemia and are in agreement with previous reports showing no protection in mice with deficiency of IL-6 in models of focal ischemia [18,19]. We observed decreased levels of KC, the murine ortholog of human IL-8 and a neutrophil chemoattractant [20], in MyD88^−/−^ mice following focal cerebral ischemia. These results concur with reports of KC production via the MyD88 pathway in a model of infection [21-23] and highlights the parallels between the innate immune response to focal ischemia, noted in our study, and the reported innate immune response to infection.

G-CSF, another neutrophil chemoattractant, which was produced in a MyD88-dependent manner in our study, has been shown to increase in the brain following cerebral ischemia in humans [24]. However, the specific downstream TLR adaptors that mediate its production following cerebral ischemia have not previously been studied. G-CSF has been reported to be neuroprotective following cerebral ischemia [25-28]. The production of G-CSF in a MyD88-dependent manner following cerebral ischemia in our current study is supported by findings of significantly reduced G-CSF levels in MyD88^−/−^ mice in models of infection [23]. The observed decrease in brain levels of G-CSF and IL-6 in MyD88^−/−^ mice, along with the reported lack of protection against cerebral ischemia in IL-6-deficient mice[19], may explain, in part, the absence of protection against cerebral ischemia in these mice reported in our previous studies [9].

The inclusion of baseline (naïve animals) cytokine/chemokine analysis in our study allowed us to show major perturbations in cytokine levels in mice that did not have surgery. This analysis permitted categorization of cytokines/chemokines into Th1/Th2 groups. Our results reveal a paradoxical Th2 phenotype, at baseline, in MyD88^−/−^ mice that reverted to a more Th1 phenotype following focal cerebral ischemia. This is in contrast to the normal switch from a Th1- to a Th2-type phenotype associated with cerebral ischemia [29]. In some cases, this abnormal baseline elevation in MyD88^−/−^ mice was found only in serum samples and, in other cases, such as IL-10, also in the brain. These findings are consistent with previous results that have shown tissue specificity in cytokine production following ischemia [30]. Interestingly, the Th2-type phenotype at baseline in MyD88^−/−^ mice is consistent with previous findings that TLRs control activation of the adaptive immune response via their role in dendritic cell maturation [31]. Importantly, these findings seem to indicate that this baseline Th2 phenotype skew in MyD88^−/−^ mice is not protective during acute cellular ischemic stress. These results may have clinical implications for patients with acute focal cerebral ischemia and an underlying chronic inflammatory condition that predisposes them to a Th2 skew at baseline, as observed in MyD88^−/−^ mice.

Overall, it is noteworthy that the cytokine profile obtained from our MyD88^−/−^ mice following cerebral ischemia was similar to the cytokine profile obtained when whole-blood cells from patients with deficiencies of MyD88 were stimulated with specific TLR agonists [32]. This highlights the physiological relevance and possible extrapolation of our current findings, with respect to the cytokine production, during the innate immune response to events occurring during cerebral ischemia in humans.

IP-10 was partially produced in a TRIF-dependent manner in our studies. However, MIP-1α was clearly produced in a predominantly TRIF-dependent manner following pMCAO, as shown by MIP-1α levels that were significantly decreased at baseline in mice with disruptions of the TRIF pathway and not in WT mice or in mice with disruptions of MyD88. Also of note, consistent with disruption of the TRIF pathway was the presence of comparable levels of IL-6 in TRIF-mutant mice and WT mice, which is evidence of a functioning MyD88 pathway. To the best of our knowledge, expression of MIP-1α has not previously been reported to occur via the TRIF signaling pathway following focal cerebral ischemia.

In this study, we have demonstrated a role for the MyD88 pathway in neutrophil migration to the site of ischemia by showing significant decreases in the levels of neutrophil chemoattractants, such as KC and G-CSF, in MyD88^−/−^ mice following focal cerebral ischemia. A study of inflammatory infiltrate with apparently fewer neutrophils in the brains of MyD88^−/−^ mice following focal ischemia provided additional evidence in this regard. These combined results strengthen our conclusion that the MyD88 pathway is involved in neutrophil migration to the site of cerebral ischemia. These findings are in agreement with studies showing involvement of the MyD88 pathway in neutrophil migration to the site of infection in the context of low levels of the neutrophil chemoattractant KC [33].

In summary, we demonstrate for the first time robust differences in cytokine production by the two different major arms of the TLR signaling pathways, MyD88 and TRIF, following focal cerebral ischemia. The significance of these findings includes the following. (1) Demonstration for the first time of new roles for the downstream TLR adaptors MyD88 and TRIF in the production of specific cytokines and chemokines in a model of focal cerebral ischemia. (2) Indications that the MyD88 pathway plays a major role in the expression of neutrophil chemoattractants, such as G-CSF and KC, which direct neutrophils to the site of tissue injury. (3) Observation of a paradoxical Th1/Th2 cytokine profile when these downstream adaptors are disrupted. These findings have not been described previously and suggest a decreased ability of these mice to mount an effective innate immune response to focal cerebral ischemia. (4) Furthermore, we describe for the first time major perturbations in the baseline cytokine profile in mice with disruptions of MyD88. These findings may explain, in part, the lack of protection against focal cerebral ischemia noted when these downstream adaptors are disrupted. Overall, our findings add to the current knowledge by describing new roles for the downstream adaptors MyD88 and TRIF following focal cerebral ischemia and providing great insights into the complex downstream TLR signaling pathway.

Some of the strengths of our study include the fact that we simultaneously tested for dynamic changes in the levels of 25 cytokines/chemokines at baseline and at different time points in brain and serum samples using multiplex array methodology. This approach allowed us to determine that the innate immune response to ischemic stress has striking similarities to the reported innate immune response to stress from infection. These similarities include the expression of cytokines and chemokines that direct the recruitment of immune cells to the site of injury as well as modulation of the Th1/Th2 response. Studies are underway to determine the differential gene expression profile in the brains of mice with disruptions of MyD88 and TRIF to help shed further light on these downstream TLR signaling events at the level of gene expression.

## Conclusions

Overall, these results provide new insights into the complex downstream TLR signaling events following cerebral ischemia. Further studies of these downstream signaling events are warranted to help identify unique control points along these pathways that may help in designing novel targeted therapies. These molecular targets may provide an approach to limit the inflammatory damage that occurs following acute cerebral ischemia, during which unbridled inflammation can exacerbate tissue damage.

## Abbreviations

IFN = Interferon; IL = Interleukin; NF-κB = Nuclear factor κB; TNF = Tumor necrosis factor; TRIF = Toll/interleukin 1 receptor domain-containing adaptor-inducing interferon β.

## Competing interests

The authors declare no competing interests.

## Authors’ contributions

BF designed the study, carried out cytokine assays and immunostaining experiments and was responsible for data analysis and interpretation as well as the writing and editing of the manuscript. YM carried out animal surgeries and reviewed the manuscript for accuracy. JH participated in study design, supervised data analysis and interpretation and critically reviewed the manuscript and approved the final version to be submitted for publication. MS contributed to data analysis and interpretation and critically reviewed and edited the manuscript. ML carried out immunostaining experiments. All authors read and approved the final manuscript.
